# Synchronising an IMX219 image sensor and AS7265x spectral sensor to make a novel low-cost spectral camera

**DOI:** 10.1016/j.ohx.2024.e00573

**Published:** 2024-08-23

**Authors:** Charles Sutherland, Alan D. Henderson, Dean R. Giosio, Andrew J. Trotter, Greg G. Smith

**Affiliations:** a School of Engineering, University of Tasmania, Dobsons Road, Sandy Bay, TAS 7005, Australia; b Institute for Marine & Antarctic Studies (IMAS), University of Tasmania, Private Bag 49, Hobart, TAS 7001, Australia

**Keywords:** Jetson Nano, AS7265x, IMX219, Low-cost, Hyperspectral, NIRS

## Abstract

A low-cost novel spectral camera able to be used for near infrared spectroscopy was made by using a Jetson Nano to synchronize a Sony IMX219 NOIR autofocus image sensor, an AMS AS7265x 18-channel spectral sensor and Osram SFH 4737 broadband infrared LED’s. Synchronizing an image sensor and spectral sensor augments a standard RGB image with light spectrum information; capturing the light distribution information normally lost in RGB image capture. Sutherland et al. [1] used this novel spectral camera to examine the dorsal surface of juvenile lobsters as a possible pre-moult detector. Having the image and spectrum in combination allowed the incomplete and unmineralized post-moult dorsal surface to be characterized with 86.7% accuracy for the first time. A proposed application for the spectral camera is to omit the local SFH 4737 light source and use the camera in daylight, effectively making a low-cost substitute hyperspectral snapshot camera. In this configuration the camera may have application for low-cost drone deployment for small scale agriculture.

## Graphical abstract

**Figure:**
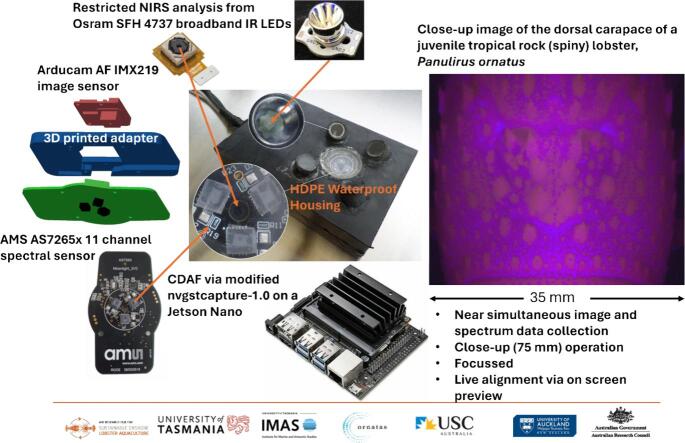

## 1 Hardware in context

Full lifecycle aquaculture production of tropical rock lobsters is a technology emerging from the Institute for Marine and Antarctic Studies (IMAS) in Tasmania. New challenges have emerged as a result, with juvenile cannibalism being problematic and causing extensive stock losses. Cannibalism is strongly associated with moulting lobsters, a process of discarding each successive exoskeleton for a new larger exoskeleton. Sutherland et al. [1] explore the possibility of using a low-cost version of near infra spectroscopy (NIRS) for pre-moult sensing based on the changed state of the pre-moult exoskeleton being disconnected from the underlying physiology of the lobster.

Research into low-cost substitution of expensive proprietary tools is being supported by a wide variety of low-cost electronics. Enabling low-cost NIRS is Osram’s SFH 4737 broadband infrared (IR) LED. ams-OSRAM AG [2] detail the background technology for SFH 4737 LEDs for consumer spectroscopy and indicate its possible inclusion on smartphones in the future. Consumer Physics Inc. [3] provide the SCiO low-cost portable NIRS scanner which uses Osram’s SFH 4737 LEDs and a proprietary sensor in a device targeted at consumer portable NIRS. Consumer Physics’ SCiO portable NIRS scanner is the closest commercial analogue to this design.

The actual sensor used in the SCiO NIRS scanner is undisclosed, however, Aquino et al. [4] use the AMS AS7265x 18-channel spectral sensor [5] in conjunction with SFH 4737 LEDs as a NIRS sensor and were able to create a low-cost tool to evaluate the fat content of olives. Noguera et al. [6] also used the SFH 4737 LED and AS7265x combination to assess the ripeness of grapes and achieved promising results. These solutions were not suitable in the case of Sutherland et al. [1] because of the need to sample lobsters with minimal disturbance in-situ. The requirement for non-contact, submerged sampling creates problems with aligning the subject at a distance and ensuring comparable data acquisition. The addition of a camera provides a real-time image for preview to allow proper alignment of the spectral camera unit and captures the scene for analysis in parallel with spectral data.

This system augments a captured image from a Sony IMX219 sensor [7] with an 18-channel spectrum from the AS7265x, and the combined data streams provide real-time alignment, mapping of key light sources in images, and an overview of the key wavelengths that contributed to the image. Key specifications and cost of the system are outlined in Table 1. The two complimentary data streams of camera image and spectral data were both required by Sutherland et al. [1] to achieve post-moult characterization from the dorsal surface of lobsters. An overarching requirement of low-cost was enforced to ensure affordability for replicated installation in commercial production facilities [1].

**Table 1:** Specifications of the novel low-cost spectral camera.

Hardware name	Novel Spectral Camera
Subject area	• General
Hardware type	• Imaging tools
Closest commercial analog	Hyperspectral snapshot camera
Open-source license	Creative Commons
Cost of hardware	$610.00
Source file repository	https://doi.org/10.5281/zenodo.13304397
OSHWA certification UID *(OPTIONAL)*	

Specific requirements for Sutherland et al. [1] were:

• Low-cost
• Submersible equipment
• Operation without contact
• Real time alignment of the target surface for comparable data shots

## 2 Hardware description

**Fig. 1:**
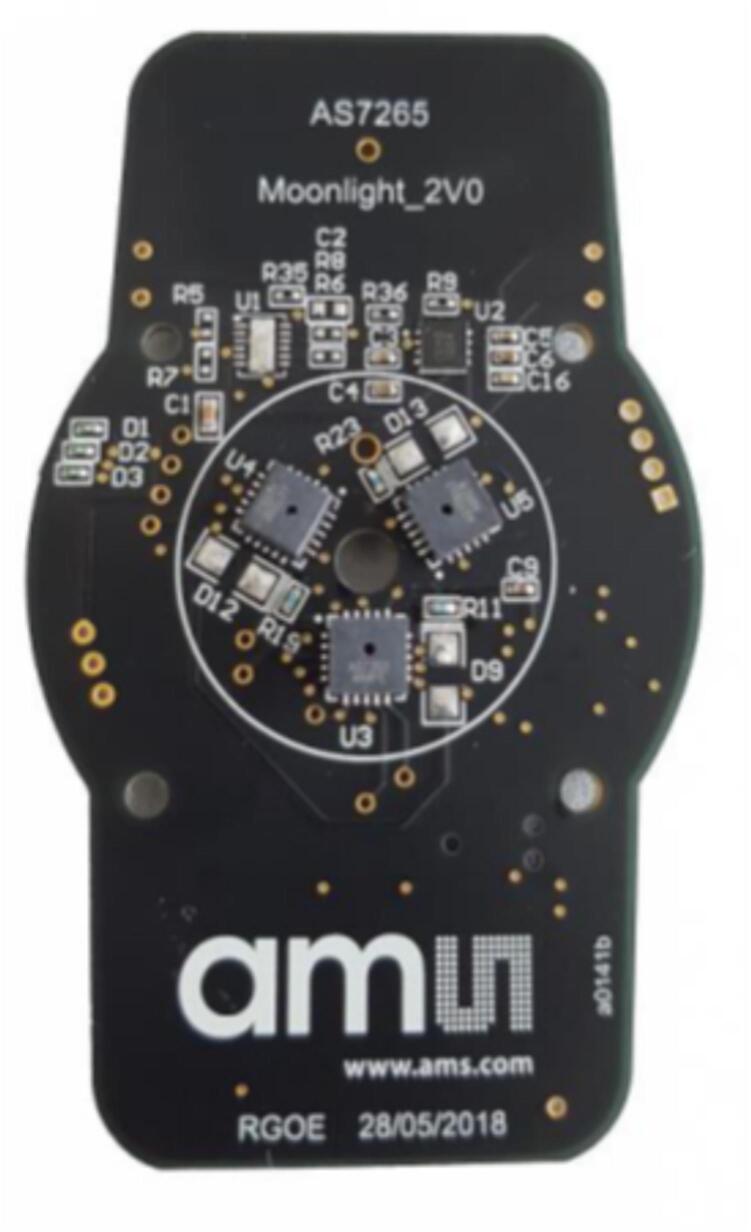
The AMS AS2765x development kit. The three individual sensors of the AS7265x family are equally distributed around a central hole.

The core hardware design is centered around a 3D printed adapter that allows an IMX219 image sensor to be aligned with a center hole in the AMS AS7265x demonstration kit development board (Fig. 1). This alignment centers the image sensor within the three individual AS7265a, AS7265b, and AS7265c spectral sensors. The central hole of the AMS AS7265x demonstration board is large enough so that the lens of the image sensor has an unobstructed view of the scene. The AMS demonstration boards also provide serial communication via a UART interface and a simple ‘AT’ command interface.

The basic spectral camera unit was extended to meet the requirements of Sutherland et al. [1] by adding SFH 4737 LEDs to create a NIRS environment. An IMX219 image sensor fitted with a motorized lens from Arducam was chosen to enable autofocusing so the system could be used at close range with an associated short depth of field [8]. The motorized lens enables short focal distance imaging and contrast detection autofocus. The Jetson Nano with its CSI/MIPI camera connectivity, powerful GPU for image processing, along with a capable GPIO and USB UART was chosen as a central coordinating and data capture system. Sutherland et al. [9] detail specialized software development for the Jetson Nano for accommodating autofocus, along with using various hardware interfaces for coordination of imaging, spectral data collection and light control. A push-button shutter release was created to allow simple, single-handed operation of the system while the other hand positioned the camera over the lobster while checking for alignment and focus on a live screen display. All data was captured with a single press of the shutter release button.

To ensure sufficient light for operation the design used four SFH 4737 LEDs. An expanded AS7265x to IMX219 adapter also aligned the SFH 4737 LEDs at the angle required to set the LEDs to focus on a point 75 mm in front of the sensors. The expanded adapter was also used to position the lid of the waterproof housing, and to ensure the sensors and camera were well aligned with an acrylic window viewport.

The four SFH 4737 LEDs were mounted in their own tubes to prevent light contamination within the housing, with the four tubes protruding past the main viewport window to ensure only reflected light was captured. The four light tubes were angled to direct the incident light to the 75 mm focal distance. The complete spectral camera was then packed into a waterproof HDPE and acrylic housing for sampling lobsters submerged in sea water.

Four IR emitting SFH 4737 LEDs create an IR radiation hazard for both the user and lobster, along with excess heat inside an enclosed housing. Addressing the IR radiation and heat problem together required SFH 4737 illumination only for data acquisition. Otherwise, an ambient LED light source was used for alignment and focusing.

The use of a camera in this design is an escalation of the systems presented by Aquino et al. [4] and Noguera et al. [6] who both use Arduino based systems which exclude the use of a synchronized camera with the AS7265x and SFH 4737 combination. A further benefit of the Jetson Nano as the system controller was effective and accurate data collection and logging [9].

An altered use case for this system is to exclude the incident lighting of the SFH 4737 LEDs and deploy as a low-cost daylight hyperspectral snapshot camera. A possible application is indicated by Moinard et al. [10] who found that the AS7265x alone could provide a reasonable Normalized Difference Vegetation Index assessment of grass cover and grape vine vigor. The successes of Moinard et al. [10] and Sutherland et al.[1] promote including the IMX219 image sensor in combination with the AS7265x to retain an image of the scene supplying light for spectral sampling. Detail of the scene captured in an image provides light mapping information, while the captured spectrum then clearly informs the distribution of light. An extension of Moinard et al. [10] may be vegetation cover assessment from a low-cost drone deployment.

Some key advantages of this system:

• Very low cost
  o Deployment to industry, attainable by smaller business
  o Low losses in high-risk deployments
  o Low-cost drone deployment
• ROV or UAV deployment
• The on-screen preview allows for accurate alignment for manual data collection.
• The motorized focus lens and autofocus provide for short distance imaging and changing focal lengths.
• Adaptable, the system is modular allowing individual components to be upgraded.

## 3 Design files

Components of this system built for purpose are presented in the design files. SpectralCamera.zip contains the parts and assemblies of the waterproof housing and are provided as an Autodesk Inventor project. LED_PCB.zip contains the design of the small heat dispersing circuit board for the SFH 4737 LEDs. Three .stl files are provided for 3D printing an adapter to mount a camera module to the AS7265x demonstration board. The adapter.stl file adapts a standard Raspberry Pi camera module V2, the adapterAF.stl is for moulting the Arducam autofocus module, and adapterHousing.stl is the carriage inside the waterproof housing that adapts the autofocus camera to the AS7265x and also aligns the four LEDs (Table 2).

**Table 2:** Design files summary.

Design file name	File type	Open-source license	Location of the file
SpectralCamera.zip	CAD	Creative commons	https://doi.org/10.5281/zenodo.13304397
LED_PCB.zip	CAD	Creative commons	https://doi.org/10.5281/zenodo.13304397
adapter.stl	CAD	Creative commons	https://doi.org/10.5281/zenodo.13304397
adapterAF.stl	CAD	Creative commons	https://doi.org/10.5281/zenodo.13304397
adapterHousing.stl	CAD	Creative commons	https://doi.org/10.5281/zenodo.13304397

## 4 Bill of materials

A comprehensive bill of materials is provided in Table 3. Global suppliers were given preference as the source of materials, although many components in this system can be substituted with available matching components.

**Table 3:** Bill of materials.

Designator	Component	Number	Cost per unit −currency	Total cost −currency	Source of material	Material type
Jetson Nano	Jetson Nano development kit	1	USD$186.25	USD$186.25	https://www.amazon.com.au/	Non-specific
Black HDPE	Waterproof Box	1	USD$30	USD$30		Polymer
Acrylic	Clear acrylic windows	5	USD$2	USD$10		Polymer
AS7265x	AS7265x demonstration kit	1	USD$208.86	USD$208.86	https://www.mouser.com/ProductDetail/ams-OSRAM/Demo-Kit-AS7265x-Multispect-chipset-v3	Semi-conductor
IMX219	IMX219 − AF image sensor (Arducam)	1	USD$22.99	USD$22.99	https://www.uctronics.com/arducam-8-mp-sony-imx219af-programmable-autofocus-noir-camera-module-for-raspberry-pi-nvidia-jetson-nano.html	Semi-conductor
¼ watt resistor	Resistor	7	USD5c	USD35c	https://www.mouser.com/c/passive-components/resistors/film-resistors/metal-film-resistors-through-hole	Non-specific
Capacitor	Capacitor	2	USD5c	USD1C	https://www.digikey.com/en/products/filter/ceramic-capacitors/through-hole/60	Non-specific
DFR0753	DFRobot	1	USD$7.90	USD$7.90	https://www.mouser.com/ProductDetail/DFRobot/DFR0753	Semi-conductor
XL 6009	DC-DC boost converter	1	USD$1.15	USD$1.15	https://www.aliexpress.com/w/wholesale-xl6009.html	Semi-conductor
2N3904 BJT	2N3904 BJTs	4	USD$0.25	USD$1.00	https://www.mouser.com/ProductDetail/Rectron/2N3904	Semi-conductor
IRF1405 MOSFET	IRF1405 MOSFET	2	USD$5.00	USD$10.00	https://www.mouser.com/ProductDetail/Infineon-Technologies/IRF1405PBF	Semi-conductor
DIODE	IN4007	2	USD1c	USD2c	https://www.mouser.com/ProductDetail/Rectron/1N4007-B	Semi-conductor
4N25 433Q	Opto coupler	1	USD71c	USD71c	https://www.mouser.com/ProductDetail/Vishay-Semiconductors/4N25	Semi-conductor
SN74HC14N	Schmitt trigger	1	USD74c	USD74c	https://www.mouser.com/ProductDetail/Texas-Instruments/SN74HC14N	Semi-conductor
Momentary switch	Momentary switch	1	USD$0.95	USD$0.95	https://www.mouser.com/ProductDetail/SparkFun/COM-11996	Non-specific
1 m HDMI cable	HDMI cable	1	USD$3.99	USD$3.99	https://au.mouser.com/ProductDetail/AAEON-UP/OPT-UP-CABLE-HDMI-001	Non-specific
FT232RL	USB UART	1	USD$1.50	USD$1.50	https://www.aliexpress.com/w/wholesale-FT232RL.html	Non-specific
UC-392	CSI to HDMI	1	USD$13.99	USD$13.99	https://www.arducam.com/product/arducam-csi-hdmi-cable-extension-module-15pin-60mm-fpc-cable-raspberry-pi-camera-specific-pack-2-1-set/	Non-specific
Reflector	OPC-11COL	4	USD$2.66	USD$10.64	https://au.mouser.com/ProductDetail/Dialight/OPC-11COL	Non-specific
LED PCB	LED PCB	4	USD$5/10 pieces	USD$10	Made to order at pcbway.com	Non-specific
STEVAL-ILL051	LED driver	2	USD$31.88	USD$63.76	https://au.mouser.com/ProductDetail/STMicroelectronics/STEVAL-ILL051V2	Non-specific
COM-13105	Cool White LED	4	USD$8.75	USD$8.75	https://www.mouser.com/ProductDetail/SparkFun/COM-13105	Semi-conductor
SFH 4737 LED	Broadband IR LED	4	USD$25.00	USD$100.00	Discontinued	Semi-conductor

## 5 Build instructions

### 5.1 Electronics construction and layout

**Fig. 2:**
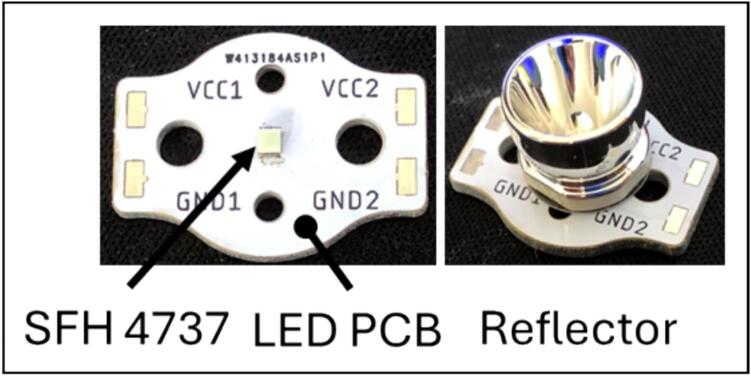
Construction of the light units. Each PCB carries a single SFH 4737 and a reflector to direct the light.

Heat dispersing aluminium PCBs for the SFH 4737 LEDs were designed and built (Fig. 2). Drill and Gerber files from LED_PCB.zip (Table 2) were provided to an online PCB manufacturer.

**Fig. 3:**
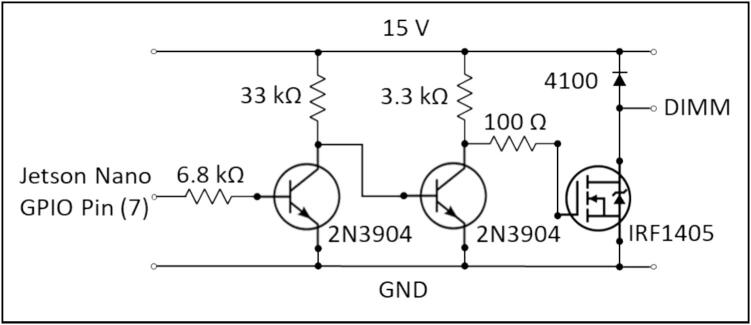
Circuit diagram for a MOSFET driver to control a LED 2000 demonstration board from a Jetson Nano GPIO pin.

**Fig. 4:**
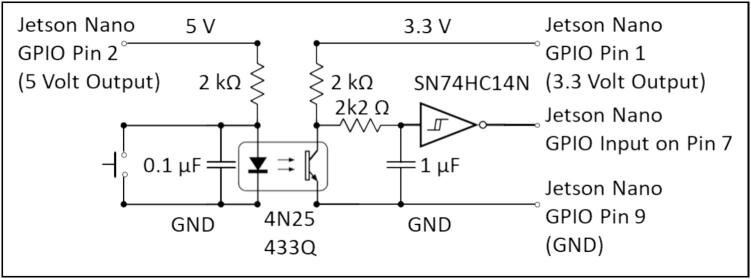
Circuit diagram for the shutter release circuit. The 4N25 433Q opto isolator filters noise from the switch cable, while the SN74HC14N Schmitt trigger debounces the input.

Two small circuit boards were constructed on perfboard as per the schematics in Fig. 3, Fig. 4. Two MOSFET drivers were built in parallel on a single piece of perfboard to drive two STEVEL-ILL051 demonstration boards with 15 Volt input (Fig. 3). The STEVEL-ILL051boards are based on STMicroelectronics’ LED2000 integrated circuit which is a dedicated LED constant current source driver with automatic voltage detection allowing strings of LEDs with varying length to be built in series and connected directly to a single driving source, with the proviso of sufficient voltage headroom. A low-cost XL 6009 DC-DC boost converter was used to boost the system’s 12 V supply to 15 V to provide voltage headroom to drive four high power LEDs in series on each of the LED circuits. The LED2000 is superseded by the LED2001 which is available on its own updated demonstration boards.

The shutter release circuit (Fig. 4) performed a dual operation of isolating noise from the GPIO header, and hardware debounce of the shutter release button. Early observations of using a switch on a long cable directly connected to the GPIO caused sever glitching of the Jetson Nano which was resolved using this circuit.

**Fig. 5:**
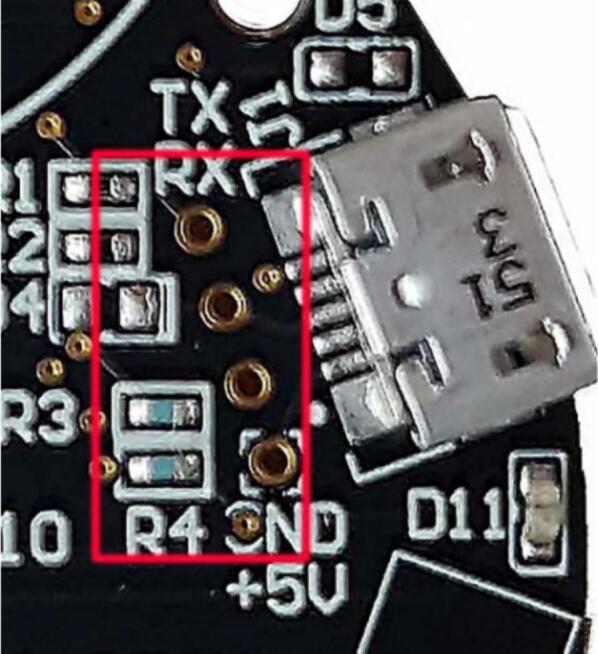
The four test points used to bypass the micro-USB port on the AS7265x (from AMS AG, 2018). Incoming wires were directly soldered as indicated by the silkscreen text at each end of the test points.

A third-party USB UART, the FT232RL set for 5-volt operation, was used as it provided reliable operation over a 1 m section of cable. The micro-USB port on the AS7265x development board was bypassed by soldering straight to the test points behind it, which also assisted in fitting the camera into the tight waterproof housing (Fig. 5).

Enabling a sufficiently long cable for a CSI/MIPI connected image sensor requires using an HDMI adapter to extend between the ends of the normally flat ribbon cable. Adapter UC-392 from Arducam was used for this purpose. The camera end of the HDMI extension adapter was accommodated within the waterproof housing.

A small piece of Tsujiden D121UP diffusion film was placed over the aperture of each AS7265x chip due to the close proximity to the reflected light source in Sutherland et al. [1]. The D121UP can be excluded when the system is sensing reflected light.

**Fig. 6:**
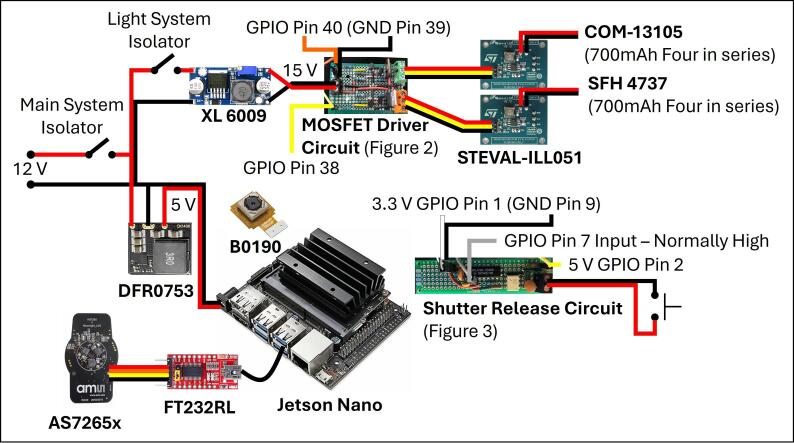
Pictorial wiring diagram for the main system components.

A pictorial schematic of wiring the individual components is shown in Fig. 6.

### 5.2 Joining the image sensor and AS7265x

**Fig. 7:**
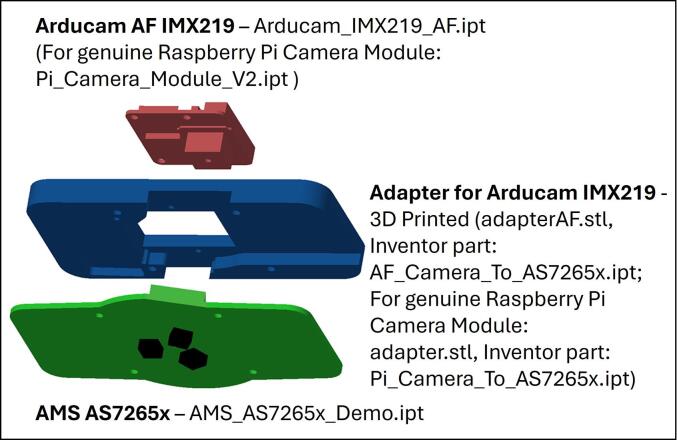
Adapting an Arducam autofocus IMX291 to align the lens of the image sensor with the centre of the AS7265x.

Three adapters are presented in stl files for 3D printing with two adapters being designed to solely attach a camera module behind the AS7265x PCB. Although the carrier boards for the Raspberry Pi camera module V2 and motorized focus IMX219 from Arducam are a similar size, the lens positioning requires differing adapters for proper lens alignment. The system shown in Fig. 7 is to adapt the Arducam autofocus version of the IMX219 image sensor. Inventor and stl files are also provided to adapt a genuine Raspberry Pi camera module V2 to the AS2765x (Table 2).

### 5.3 The waterproof HDPE housing

Construction of the HDPE waterproof housing will vary depending on local workshop capabilities. Inventor design files are in SpectralCamera.zip (Table 2). The housing used in Sutherland et al. [1] was made on traditional lathes and milling centers. The acrylic windows were laser cut. The light tubes and clear acrylic windows were glued into the HDPE housing using a polyolefin primer (Loctite SF770) and cyanoacrylate glue. This primer and glue provided firm, water-tight bonds between HDPE to HDPE and HDPE to acrylic. It is expected that a capable 5-axis CNC machining center could make the housing base with the light tubes incorporated as single piece assembly.

The housing was required for surface use only. Testing the housing for pin hole leaks through the glued joints involved making a water-tight skirt to allow the main housing section with the glued joints to be submersed to a depth of 100 mm. The two parts of the housing were carefully glued together using Sikaflex 291 Marine Adhesive Sealant (black polyurethane). Finally, the joint was taped with PVS duct tape. The housing was regularly inspected for ingress of water during use.

**Fig. 8:**
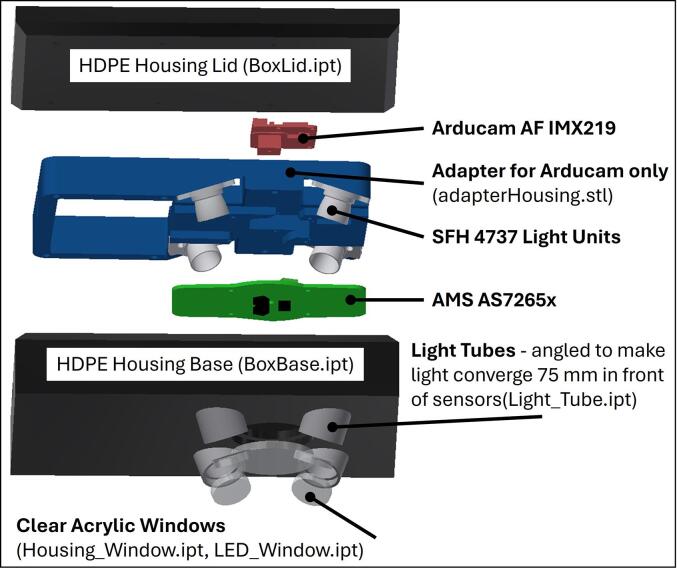
Construction of the spectral camera and SFH 4737 light units within an HDPE waterproof housing.

The adapterHousing.stl file is for a larger carrier/adapter designed for use in the HDPE waterproof housing and caters for the Arducam autofocus image sensor alone (Table 2). The adapter is fitted and wired for the AS7265x, IMX219 and four SFH 4737 light units before being screwed to the housing lid. The housing lid and adapter unit is then inserted into the housing base, carefully placing the SFH 4737 light units into the light tubes. The adapter is a sliding fit into the housing base and serves to align the system components with the light tubes and windows (Fig. 8).

### 5.4 Software

The software is described in a companion article in SoftwareX and is presented separately because its development is the greater component in this system’s development. The software is developed specifically for use on the Jetson Nano but should be adaptable to other Jetson modules. The software builds on a C coded Jetson camera application called Nvgstcapture-1.0 and provides autofocus for the camera and UART control of the AS7265x demonstration board. The provided UART interface simplified software development for the development of this low-cost camera, and along with the presence of a hole central to the placement of the three AS7265x sensors, determined that the more expensive AMS AS7265x demonstration board was most suited to development of this prototype.

### 5.5 Safety

• Cyanoacrylate glues can rapidly join skin. The use of activators can result in rapidly setting glue and can generate enough heat to burn skin.
• Ensure machine operation is done by suitably qualified persons.
• Soldering fumes and heat are hazards associated with building custom circuit boards.

## 6 Operation instructions

• Ensure the correct data and time is set on the Jetson Nano, especially if not connected to the Internet.
• Ensure the light system isolation switch remains off until the control application is running.
• Run the extended nvgstcapture-1.0 application (Sutherland et al. [9]).
• A live camera image appears in its own window and the terminal message logs indicate that hardware interfaces have been set up.
• Power-on the light system circuit.
• Align the camera with the target using the live on-screen preview.
• Wait for focus and press the shutter release.
• Generation of a directory named with the current date is automatic.
• Data files for the captured image (JPG) and AS7265x spectral data (TXT) are generated and named with the time the shutter release was pressed.
• Continue data capture with repeated presses of the shutter release.
• Exposure to IR radiation can be hazardous, ensure shielding is positioned between the camera operation and the preview screen. The operator should not observe the camera whilst in operation.

## 7 Validation and characterization

Sutherland et al. [1] report on the operation of the SFH 4737 LEDs when driven by the STEVAL-ILL051V1 board and showed that readings taken with a SpectralEvolution RS-8800 spectrometer off a 127x127 mm (5x5 inch) white 99 % Spectralon reflectance panel was a close reproduction of performance figures from the SFH-4737 datasheet [11]. The SFH 4737 LED’s emitted approximately equally through their specified broadband IR wavelengths between 760 nm and 1050 nm.

In application for Sutherland et al. [1] the spectral camera was stable and reliable, had near simultaneous (<100 ms) image and spectral data capture. Repeatable data acquisition was a noted characteristic of this system. Having a camera and preview feed successfully allowed the lobster carapace to be aligned to acquire comparative data, with the autofocus being adequate to allow use at close range. The development of this low-cost camera met low-cost requirements of industry in its prototype form, however scope for further reducing costs exists. The circuit driving the DIMM pin for LED on/off control via the STEVAL-ILL051V1 could be simplified, potentially down to a single BJT transistor. This could be further supported by configuring the LED driver IC (the STEVAL-ILL051V1 uses the now superseded LED2000) to be set at a higher current allowing the LED array to operate in parallel and thus reduce the maximum system voltage required. The XL6009 boost converter could be excluded from the design. The greatest saving could be achieved by developing a custom carrier board to carry both the image sensor and three AS7265x spectral sensors. The individual AS7265x sensors are available for between USD$6 to $13 providing a significant saving if demand arises.

Along with meeting low-cost requirements for industry, the primary impetus for the development of this camera was to determine whether it had any ability to act as a pre-moult sensor [1]. A patent granted to Garland and Sakretta [12], uses infrared light and a changing appearance on the dorsal carapace of nephropid (e.g. *Homarus americanus*) lobsters to relate to moult stage progress, which further relates to meat density and lobster quality. The biological changes that occurs to the exoskeleton of palinurid lobsters at pre-moult are extensive, and it is was not considered unreasonable that a biochemical marker detectable under infrared light may exist [1]. A thorough search of the literature did not reveal any attempt to find such a marker. The USD $20,000 price for a hyperspectral snapshot camera prohibited an exploratory study under the conditions of high uncertainty of Sutherland et al. [1], where key parameters were unknown. However, the $610 dollar price tag of this low-cost camera did not impede an exploratory study based solely on the speculation that an extensive difference may exist between carapaces of intermoult and pre-moult palinurid lobsters when sampled in an IR environment. Meeting the low-cost requirement from industry excluded more refined higher-grade tools. If a pre-moult change was not sufficiently distinct on the exoskeleton to enable its detection with this low-cost camera, then a pre-moult sensor using an IR environment would be unsuitable for industrial application overall.

The specific nature of the test application for this camera exposes some drawbacks for full validation and characterization of the camera. The methodology presented in Sutherland et al. [1] was entirely based on comparing raw data gathered from lobsters already classified into three clearly defined test groups. Detailed calibration of the camera’s components was not required and so cannot be presented here. Combining the spectral and image data streams using a binary classification decision boundary on a 2-dimensional plot of the summed infrared channel data, and the percentage of bright pixels on the dorsal carapace surface based on a threshold operation in images, is a method that is unlikely to have application outside of the particular case of Sutherland et al. [1]. However, the potential for this low-cost camera to have wider application is evident and it is not supposed that the parameters of operation by Sutherland et al. [1] will suit the camera’s application in other research endeavors.

The spectral camera’s low-cost makes it ideal as a general-purpose tool with wide application potential as a low-cost substitute for a hyperspectral snapshot camera. Foregoing per pixel spectral readings limiting the scope of application, where the acquired image could be considered mostly uniform, a single spectral reading may suffice. Another application is when using CMOS image sensors for infrared image sensing. Typically, the RGB channels, identified because they have different sensitivities in the red, green and blue regions of the color spectrum, have a single overlying peak sensitivity in the IR region of the sensitivity when the IR reject filter is removed in front of the sensor. Infrared imaging with a typical RGB CMOS sensor is unable to capture any IR spectral information, a limitation that is overcome using the ability of this camera to simultaneously capture the IR spectral data.

There is an expectation that this camera, or a variant of it using substituted components, will have broad application. The expectation is based on the wide application of cameras in general, and the broadening application of hyperspectral cameras in research, industrial and agricultural pursuits [13], [14], [15], [16]. For this reason, the camera is presented as ready to be used in a variety of applications, which will have specific calibration and data analysis requirements that will be application specific.

The literature provides support for the seemingly premature presentation of this low-cost spectral camera as having much wider application potential than the example application of Sutherland et al. [1]. The individual major components of this system, the IMX219 and the AS7265x are well characterized in the literature. Characterization of the IMX219 image sensor is thoroughly documented by Pagnutti et al. [17] who conclude that the IMX219 is suitable for use in scientific applications. More importantly, the literature reports a variety of uses of the AS7265x sensor. Botero-Valencia and Valencia-Aguierre [18] provide a comprehensive characterization of the AS7265x, and Leon-Salas et al. [19] present a comprehensive analysis and calibration technique for the AS7265x for the purpose of measuring photosynthetically active radiation (PAR). Use of the AS7265x in conjunction with SFH 4737 LEDs as a NIRS sensor is characterized and validated by Aquino et al. [4] and Noguera et al. [6].

Moinard at al. [10] report that the AS7265x is capable of grass cover and grape vine rigor assessment after individual unit calibration to the task. The addition of an image sensor to provide scene mapping may benefit this application and suggests other potential applications for the spectral camera. Excluding the incorporated SFH 4737 incident light source frees the camera’s core components, the IMX219 and AS7265x, to be tested as a low-cost hyperspectral snapshot camera for vegetation mapping.

Each of these applications detail differing calibration and data analysis approaches which suit the application that the AS7265x is being used for. It is expected that flexible use of the AS7265x demonstrated in the literature will carry over and be extended as a component in this low-cost camera. Similarly, merging the image and spectral data streams will require flexibility to adapt to new applications for this low-cost spectral camera.

The low-cost novel spectral camera is adaptable, with Sutherland et al. [1] demonstrating its ability as a NIRS system under illumination from SFH 4737 LEDs. Extensive investigation and validation of individual components in the literature all indicate that with proper calibration and coordination, the multiple data streams of image and an 18-channel spectrum provide flexibility to adapt to per application requirements. With applicability ranging from close range imaging through aquatic environments to possible vegetation mapping, this spectral camera suits a variety of potential applications.

### 7.1 Capabilities and performance

• Using the hardware debounce/filter circuit on the shutter release button is critical for smooth operation. The Jetson Nano GPIO header is sensitive to electrical noise.
• The SFH 4737 lights are viable over only short distances and are aligned to point 75 mm in front of the AS7265x spectral sensor to ensure adequate IR light saturation.
• Light intensity fluctuations occur if the subject is off center, or the distance between camera and subject varies.
• Camera images augmented with spectral data may have uses beyond a low-cost substitute for a NIRS system.
• Use of spectral camera unit under natural light creates a low-cost, reduced capability, hyperspectral snapshot camera which may suit low-cost deployment on drones for vegetation mapping.

## Ethics statement

Ethical review and approval were waived for this study due to research on invertebrates is currently exempt from ethics approval as per the current Australian code for the care and use of animals for scientific purposes. “The Code applies to the care and use of all live non-human vertebrates and cephalopods.” Decapod crustaceans do not fall within these categories.

## CRediT authorship contribution statement

**Charles Sutherland:** Writing – original draft, Software, Conceptualization, Investigation. **Alan D. Henderson:** Writing – review & editing, Supervision, Resources, Project administration, Funding acquisition. **Dean R. Giosio:** Writing – review & editing, Supervision. **Andrew J. Trotter:** Writing – review & editing, Supervision. **Greg G. Smith:** Writing – review & editing, Supervision, Resources, Project administration, Funding acquisition.

## Declaration of competing interest

The authors declare that they have no known competing financial interests or personal relationships that could have appeared to influence the work reported in this paper.
